# Oligosaccharides Ameliorate Acute Kidney Injury by Alleviating Cluster of Differentiation 44-Mediated Immune Responses in Renal Tubular Cells

**DOI:** 10.3390/nu14040760

**Published:** 2022-02-11

**Authors:** Tso-Hsiao Chen, Chung-Te Liu, Chung-Yi Cheng, Yuh-Mou Sue, Nai-Jen Huang, Cheng-Hsien Chen

**Affiliations:** 1 Division of Nephrology, Department of Internal Medicine, Wan Fang Hospital, Taipei Medical University, Taipei 116, Taiwan; tsohsiao@tmu.edu.tw (T.-H.C.); 96320@w.tmu.edu.tw (C.-T.L.); 94426@w.tmu.edu.tw (C.-Y.C.); sueym@tmu.edu.tw (Y.-M.S.); milkmilklen@gmail.com (N.-J.H.); 2 Department of Internal Medicine, School of Medicine, College of Medicine, Taipei Medical University, Taipei 110, Taiwan; 3 TMU Research Center of Urology and Kidney, Taipei 110, Taiwan; 4 Division of Nephrology, Department of Internal Medicine, Shuang Ho Hospital, Taipei Medical University, New Taipei City 235, Taiwan

**Keywords:** acute kidney injury, cluster of differentiation 44, fructo-oligosaccharide, galacto-oligosaccharide, oligo-fucoidan, inflammation

## Abstract

Acute kidney injury (AKI) is a sudden episode of kidney damage that commonly occurs in patients admitted to hospitals. To date, no ideal treatment has been developed to reduce AKI severity. Oligo-fucoidan (FC) interferes with renal tubular cell surface protein cluster of differentiation 44 (CD44) to prevent renal interstitial fibrosis; however, the influence of oligosaccharides on AKI remains unknown. In this study, FC, galacto-oligosaccharide (GOS), and fructo-oligosaccharide (FOS) were selected to investigate the influence of oligosaccharides on AKI. All three oligosaccharides have been proven to be partially absorbed by the intestine. We found that the oligosaccharides dose-dependently reduced CD44 antigenicity and suppressed the hypoxia-induced expression of CD44, phospho-JNK, MCP-1, IL-1β, and TNF-α in NRK-52E renal tubular cells. Meanwhile, CD44 siRNA transfection and JNK inhibitor SP600125 reduced the hypoxia-induced expression of phospho-JNK and cytokines. The ligand of CD44, hyaluronan, counteracted the influence of oligosaccharides on CD44 and phospho-JNK. At 2 days post-surgery for ischemia–reperfusion injury, oligosaccharides reduced kidney inflammation, serum creatine, MCP-1, IL-1β, and TNF-α in AKI mice. At 7 days post-surgery, kidney recovery was promoted. These results indicate that FC, GOS, and FOS inhibit the hypoxia-induced CD44/JNK cascade and cytokines in renal tubular cells, thereby ameliorating AKI and kidney inflammation in AKI mice. Therefore, oligosaccharide supplementation is a potential healthcare strategy for patients with AKI.

## 1. Introduction

Acute kidney injury (AKI) is characterized by a sudden decrease in glomerular filtration rate and a consequent increase in serum creatinine. This damage is common in patients admitted to hospitals and intensive care units, especially the elderly. Ischemia, sepsis, and nephrotoxicity are the main causes of AKI that often co-exist and, thus, complicate diagnosis and treatment [1]. Severe AKI places discharged patients at risk of adverse events, such as chronic kidney disease, cardiovascular events, cancer, and infections [2]. AKI has a rapid development, and no effective treatment is available to avoid or reduce its severity. Intake of specific nutrients can reduce renal tubular injury in AKI [3]. Therefore, studying the influence of specific nutrition on AKI will aid in ameliorating AKI and accompanying risks faced by discharged patients.

Inflammation and leukocyte recruitment are key mediators of AKI. Kidneys, especially renal tubular cells, generate inflammatory cytokines during AKI, such as IL-1, IL-6, monocyte chemoattractant protein-1 (MCP-1), and tumor necrosis factor-α (TNF-α) [4,5]. These cytokines further induce systemic inflammation and leukocyte recruitment. Although several leukocyte subsets exert protective effects on AKI (such as T-regulatory cells), most neutrophils, monocytes, and dendritic cells are harmful to AKI recovery [6]. Systemic inflammation is also detrimental to recovery from AKI because of the high cytokine levels [7]. Among the cytokines secreted by the kidney, MCP-1 is mainly responsible for leukocyte recruitment, responds to various cytokines, including IL-1α and TNF-α [8,9], and plays an important role in the pathogenesis of different kidney injuries [10]. In human proximal tubular cells HK-2, hypoxia-induced MCP-1 expression is mediated by the c-Jun NH2-terminal kinase (JNK) pathway [11]. The p38 pathway is also involved in albumin-induced MCP-1 expression in renal tubular epithelial cells [12]. Therefore, the regulation of JNK and p38 pathways may influence inflammation and leukocyte recruitment in AKI.

Oligosaccharides are saccharide polymers containing 3–10 monosaccharides that are indigestible and constitute a major component of total available prebiotics as dietary additives [13]. Most fructo-oligosaccharides (FOSs) ingested in the human body are completely fermented by colonic flora, and a small fraction is recovered in urine [14]. Galacto-oligosaccharides (GOSs) can be absorbed in the blood and subsequently excreted in urine [15]. The same situation applies to fucoidan in brown marine algae [16]. Absorbed oligosaccharides can influence biochemical reactions and cell recognition [17]. Our previous study reveals that oligo-fucoidan (FC) interferes with the interaction between the renal tubular cell surface protein CD44 and its extracellular ligands, thereby preventing renal interstitial fibrosis [18]. The interaction between CD44 and its extracellular ligands promotes the JNK signaling pathway [19]. Therefore, the daily ingestion of oligosaccharides might reduce kidney inflammation in patients with AKI. However, the influence of these polymers on AKI remains unknown. This study investigated the effect of FC, FOS, and GOS on hypoxia-treated renal tubular cells and AKI mice with ischemia–reperfusion injury (IRI). Results showed that the three oligosaccharides suppressed CD44-mediated renal immune responses and reduced post-AKI severity. Therefore, the use of these polymers as a nutritional supplement presents a new AKI treatment strategy.

## 2. Materials and Methods

### 2.1. Materials

FOS (average degree of polymerization < 10), GOS (average degree of polymerization < 8), and FC (average degree of polymerization < 5) were obtained from Sigma-Aldrich (St. Louis, MO, USA, Cat. No. F8052), Carbosynth (Berkshire, UK, Cat. No. OG32134), and Hi-Q Marine Biotech International (Taipei, Taiwan), respectively. Oligosaccharides were dissolved in double-distilled H_2_O and filtered using 0.22 μm sterile filters (Merck Millipore, Darmstadt, Germany). Hyaluronan (HA) and all other chemicals of reagent grade were obtained from Sigma. Antibodies against JNK (sc-7345), phospho-JNK (sc-6254), MCP-1 (sc-52701), IL-1β (sc-12742), and TNF-α (sc-52746) were purchased from Santa Cruz Biotechnology (Dallas, TX, USA). Antibodies against p38 (9212) and phospho-p38 (9211) were obtained from Cell Signaling Technology (Danvers, MA, USA). Antibodies against Ly6G (ab25377) and F4/80 (ab100790) were purchased from Abcam (Cambridge, UK). Antibodies against HIF-1α (NB100-105) were purchased from Novus Biologicals (Centennial, CO, USA).

### 2.2. Cell Culture

Rat proximal renal tubular cells (NRK-52E) and mouse macrophage line RAW264.7 were purchased from the Food Industry Research and Development Institute (Hsinchu, Taiwan); cultured in DMEM supplemented with antibiotic/antifungal solution and 10% fetal calf serum; and kept at 37 °C and 95% humidity in a CO_2_ incubator. After reaching 70% confluence, the cells were cultured in serum-free medium and incubated overnight prior to the experiment. For hypoxia experiment, NRK-52E cells and Anaero Pack (Mitsubishi Gas Chemical America, New York, NY, USA) were deposited into rectangular jars (Mitsubishi Gas Chemical America), and the entire setup was placed in a 37 °C incubator for 4 h. The cells were then cultured overnight under normal conditions.

### 2.3. Western Blot Analysis

In brief, 15 µg of NRK-52E protein lysate was applied to each lane, and the results were analyzed by Western blot. Relative protein band levels were quantified from five independent experiments by Quantiscan software from Biosoft (Cambridge, UK).

### 2.4. Short Interfering (si)RNA Transfection of CD44

Rat CD44 siRNA (4390771) was purchased from Thermo Scientific (Waltham, MA, USA). After growing to 70% confluence, the cells were transfected with siRNA using Lipofectamine 2000 transfection reagent (Thermo Scientific) in accordance with the manufacturer’s instructions.

### 2.5. Cell Proliferation Assay

After hypoxic NRK-52E cells were cultured for 24 h, the same medium was used to culture RAW264.7 cells in a 96-well plate (2000 cells/well). After 24 h of incubation, the proliferation of RAW264.7 cells was analyzed by using an SRB Assay Kit (Abcam, Cat. No. ab235935) in accordance with the manufacturer’s instructions.

### 2.6. CD44 Antigenicity Tests

Recombinant rat CD44 was obtained from R&D Systems (Minneapolis, MN, USA, Cat. No. 6577-CD) and employed to produce a binding reaction with HA in accordance with the manufacturer’s instructions. In brief, 20 ng/mL recombinant rat CD44 was mixed with different concentrations of FC, FOS, or GOS for 10 min to investigate the influence of oligosaccharides on CD44 antigenicity. The mixtures were analyzed using CD44 ELISA kit (Abcam, Cat. No. ab213928) in accordance with the manufacturer’s instructions.

### 2.7. Experimental Animals and Renal IRI

Eight-week-old male 129S1/SvImJ mice were purchased from Lasco Technology (Taipei, Taiwan). All animal experiments were approved by the Taipei Medical University Committee of Experimental Animal Care and Use (approval No. LAC-2019-0511) and performed in accordance with the NIH Guide for the Care and Use of Laboratory Animals. For IRI induction, mice (weighing 21–23 g) were subjected to bilateral renal pedicle clamping for 30 min by conducting flank incision [20]. Mice were anesthetized by inhalant anesthesia with isoflurane preoperatively. Sham-operated mice underwent a similar operation, except for the clamping of renal pedicles. All mice were fed with oligosaccharides through oral gavage from 1 day before to 2 days after IRI surgery. The experimental mice were divided into the following five groups with n = 8 for the results on day 3 and day7: (1) sham group; (2) AKI group; (3) AKI group fed with FC (10 mg/kg/day); (4) AKI group fed with FOS (10 mg/kg/day); and (5) AKI group fed with GOS (10 mg/kg/day). Mice were sacrificed 3 or 7 days after the surgery, and blood was collected and analyzed using biochemical analyzer and ELISA kits. The serum creatinine level of each mouse was analyzed to assess the development of AKI. Kidney morphology was examined by periodic acid–Schiff (PAS) staining and immunohistochemistry staining (IHC) in formalin-fixed 2 μm paraffin sections.

### 2.8. Immunohistochemistry Staining

Kidney tissues were fixed with 4% buffered paraformaldehyde, embedded in paraffin, and cut into 2 μm sections for staining with UltraVision Quanto Detection System HRP DAB kits (Thermo Scientific, Fremont, CA, USA) and hybridizing with the prime antibody in accordance with the manufacturer’s instructions.

### 2.9. PAS Staining

Kidney sections were stained with a PAS Stain Kit (Abcam, Cat. No. ab150680) in accordance with the manufacturer’s instructions.

### 2.10. ELISA Assay

The cultured medium of NRK-52E cells and the serum samples of the experimental mice were collected and analyzed using the MCP-1 ELISA kit (Abcam, Cat. No. ab208979) and IL-1β ELISA kit (Abcam, Cat. No. ab197742). The serum samples were also analyzed using TNF-α ELISA kit (R&D Systems, Cat. No. MTA00B) and neutrophil gelatinase-associated lipocalin (NGAL) ELISA kit (R&D Systems, Cat. No. MLCN20) in accordance with the manufacturer’s instructions.

### 2.11. Statistical Tests

Significant differences between two groups were determined using Student’s *t*-test. Differences were considered significant for *p* values <0.05.

## 3. Results

### 3.1. Oligosaccharides Inhibit the Increase in CD44 and Cytokines in Hypoxic NRK-52E Cells

**Figure 1:**
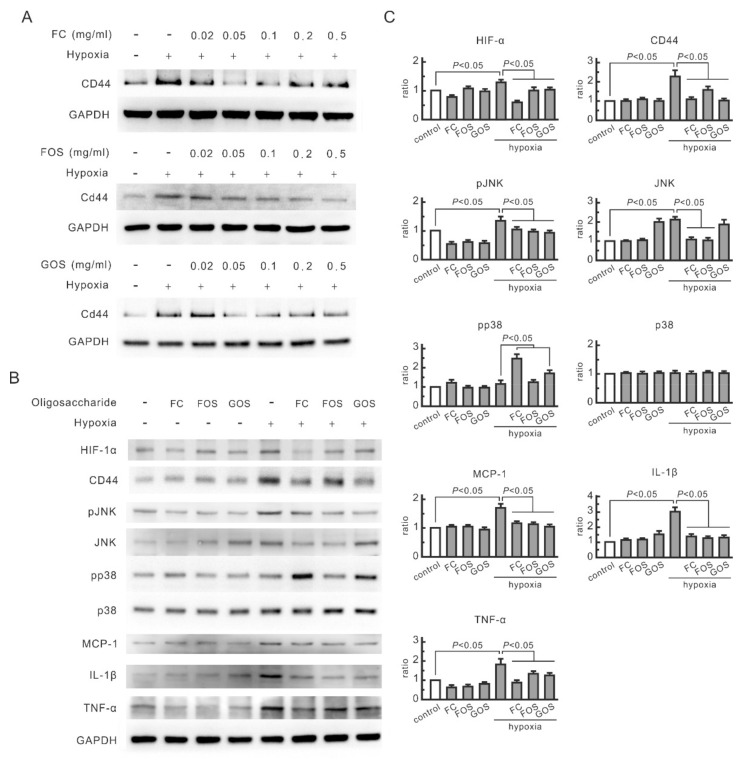
Inhibitory effect of oligosaccharides on CD44 signal transduction in hypoxic NRK-52E cells. The cells were pretreated with FC, FOS, or GOS for 30 min and then subjected to hypoxic culture. Protein expression was analyzed by Western blotting. (**A**) Dose-dependent effects of oligosaccharides on CD44 expression. (**B**) Effect of oligosaccharides on CD44-related signals. Oligosaccharide concentration was set as 0.1 mg/mL. Relative increases in protein bands are also presented in bar chart form (**C**). Results are expressed as mean ± SD (n = 4).

CD44 expression in NRK-52E cells treated with FC, FOS, and GOS was first monitored to evaluate the influence of oligosaccharides on renal tubular cells. Hypoxia-induced CD44 expression was inhibited by all three oligosaccharides in the range of 0.05–0.5 mg/mL (Figure 1A). Therefore, the oligosaccharide dosage of 0.1 mg/mL was used in the following cell experiments. In the prepared system, hypoxia treatment remarkably induced the expression of hypoxia marker HIF-1α in NRK-52E cells, indicating that the cells were in a hypoxia state (Figure 1B). Hypoxia upregulated the levels of phosphorylated JNK, MCP-1, IL-1β, and TNF-α but not in the cells treated with oligosaccharides (Figure 1B,C). JNK expression increased in hypoxic cells but not in FC-treated or FOS-treated cells. GOS did not affect JNK expression in hypoxic cells but suppressed the upregulation of phosphorylated JNK. Therefore, oligosaccharides can inhibit hypoxia-induced JNK signaling transduction and cytokine expression in renal tubular cells. Although FC and GOS upregulated phosphorylated p38 in hypoxic cells, the expression of this protein did not increase in hypoxic cells. This finding indicates that p38 does not play a key role in hypoxia-induced responses in NRK-52E cells.

### 3.2. CD44 and JNK Play a Critical Role in Cytokine Increase in Hypoxic NRK-52E Cells

**Figure 2:**
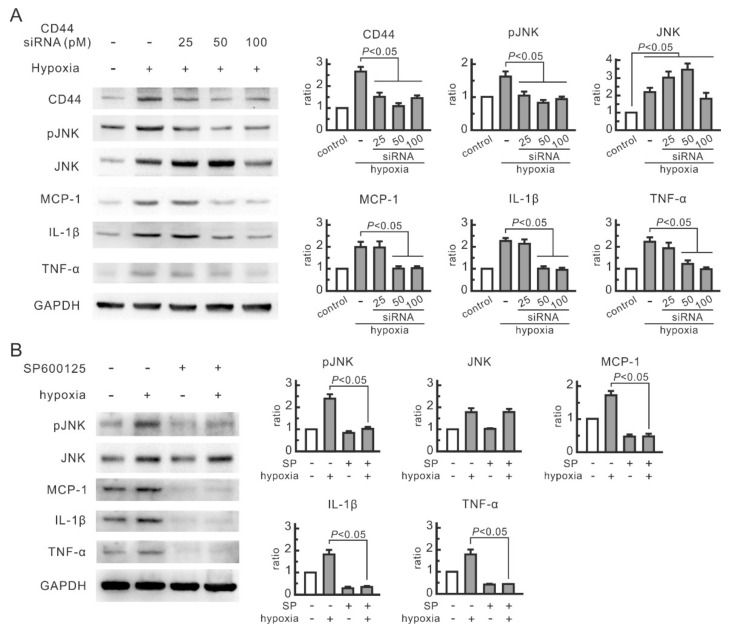
Role of CD44 and JNK in hypoxia-induced inflammatory responses in NRK-52E cells. Protein expression was analyzed by Western blot. (**A**) Inhibitory effect of CD44 siRNA transfection on phosphorylated JNK and cytokines. (**B**) Inhibitory effect of SP600125 on cytokines. The cells were pretreated with 20 μM SP600125 for 30 min prior to hypoxia treatment. Relative increases in protein bands are also presented in bar chart form. Results are expressed as mean ± SD (n = 4).

The expression of CD44, phosphorylated JNK, and cytokines in renal tubular cells were all affected by oligosaccharides, but their association remains to be elucidated. In order to investigate the role of CD44 in JNK signaling transduction and cytokine expression in hypoxic cells, NRK-52E cells were transfected with different doses of CD44 siRNA. CD44 siRNA at 50 and 100 pM efficiently blocked hypoxia-increased CD44 expression and significantly inhibited hypoxia-induced increase in the levels of phosphorylated JNK, MCP-1, IL-1β, and TNF-α (Figure 2A). The influence of CD44 blocking on JNK expression was related to siRNA doses. JNK expression in hypoxic cells was induced by CD44 siRNA at 50 pM but inhibited at 100 pM. Both doses suppressed the upregulation of phosphorylated JNK. The selective inhibitor for JNK, SP600125, significantly reduced the expression of MCP-1, IL-1β, and TNF-α in hypoxic NRK-52E cells (Figure 2B). These results suggest that the inhibitory effect of CD44 siRNA transfection on hypoxia-induced cytokines is attributed to a reduction in phosphorylated JNK.

### 3.3. Hypoxia-Induced Cytokine Secretion in Renal Tubular Cells Promotes Macrophage Proliferation

**Figure 3:**
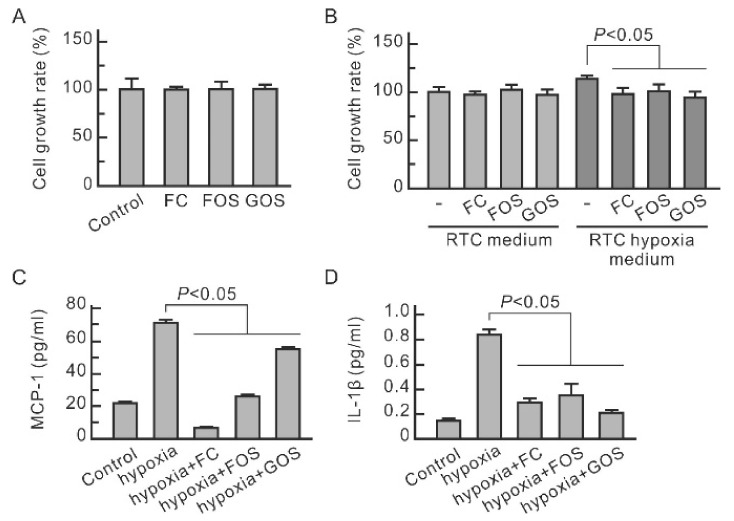
Promoting effect of cytokines secreted from hypoxic NRK-52E cells on the proliferation of macrophage RAW264.7. (**A**) Influence of oligosaccharides on the proliferation of RAW264.7 cells treated with 0.1 mg/mL FC, FOS, or GOS and cultured overnight. (**B**) Promoting effect of the culture medium of hypoxic NRK-52E cells on the proliferation of RAW264.7 cells. NRK-52E cells were pretreated with 0.1 mg/mL FC, FOS, or GOS for 30 min and then subjected to hypoxic culture. The culture medium of normal or hypoxic NRK-52E cells was taken out and used to culture RAW264.7 overnight. (**C**) MCP-1 level in the culture medium of hypoxic NRK-52E cells. (**D**) IL-1β level in the culture medium of hypoxic NRK-52E cells. Results are expressed as mean ± SD (n = 3).

Inflammation is a mediator of renal injury in AKI. Hence, the influence of oligosaccharides on leukocytes was examined. Figure 3A shows that FC, FOS, and GOS did not influence the proliferation of macrophage RAW264.7 cells. The proliferation of RAW264.7 cells was promoted by the 24 h culture medium for hypoxic NRK-52E cells (Figure 3B) but not by the medium for hypoxic NRK-52E cells treated with oligosaccharides. The former had higher MCP-1 and IL-1β levels than the latter (Figure 3C,D). All three oligosaccharides significantly reduced MCP-1 and IL-1β in the culture medium of hypoxic NRK-52E cells. Therefore, FC, FOS, and GOS can reduce the expression and secretion of cytokines in hypoxic renal tubular cells and, consequently, diminish inflammation caused by injured renal tubular cells.

### 3.4. Oligosaccharides Reduce CD44 Antigenicity

**Figure 4:**
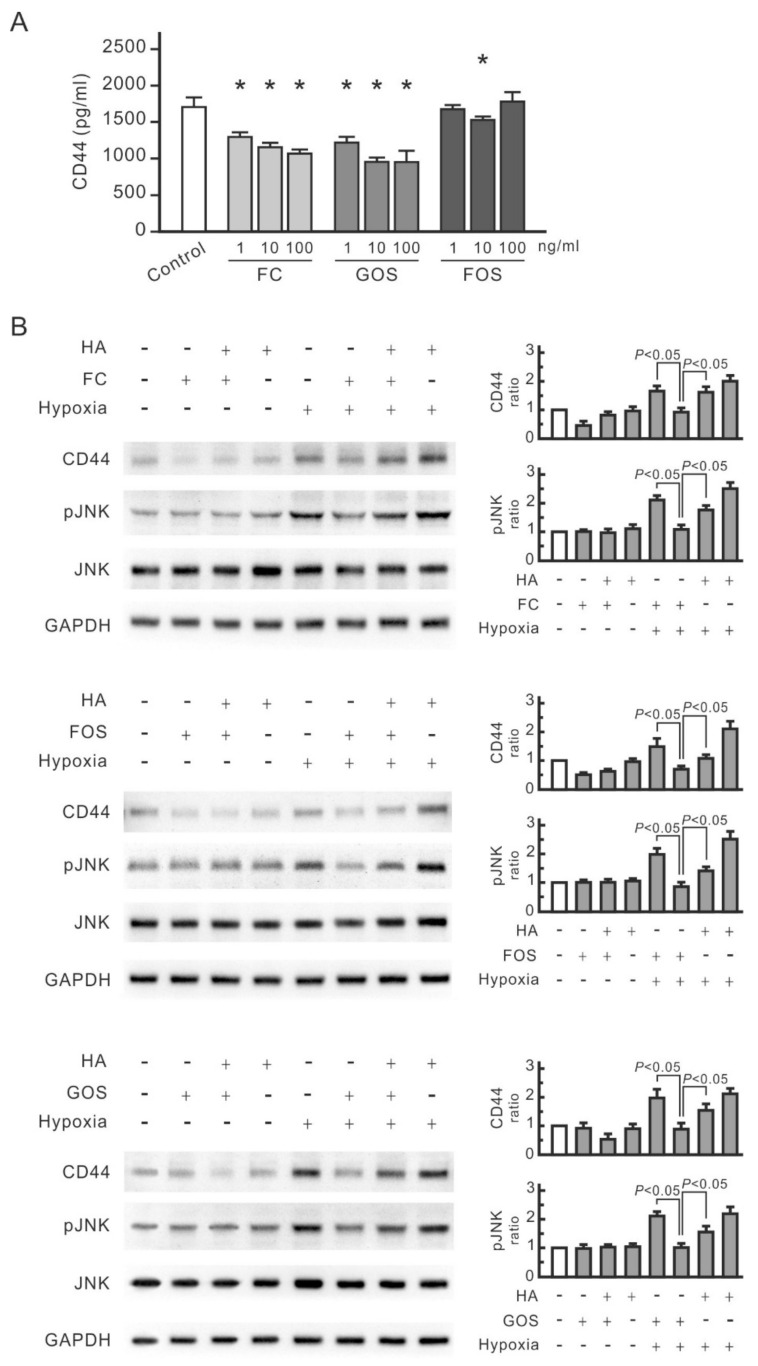
Competitiveness of oligosaccharides and HA in binding to CD44. (**A**) Reducing effect of oligosaccharides on CD44 antigenicity. Recombinant rat CD44 (20 ng/mL) was mixed with or without FC, FOS, or GOS for 10 min and then analyzed using the CD44 ELISA kit. Results are expressed as mean ± SD (n = 4). *, *p* < 0.05 vs. the control group. (**B**) HA interfering with the inhibitory effect of oligosaccharides on CD44 and phosphorylated JNK expression. NRK-52E cells were pretreated with 0.1 mg/mL HA for 30 min, then administered with 0.1 mg/mL FC, FOS, or GOS for 30 min and finally subjected to hypoxic culture. Protein expression was analyzed by Western blot. Relative increases in the protein bands are also presented in bar chart form. Results are expressed as mean ± SD (n = 4).

Given that FC interferes with the interaction between CD44 and its ligands, such as HA and osteopontin, a potential interaction might occur between oligosaccharides and CD44. For confirmation, the influence of FC, FOS, and GOS on CD44 antigenicity was examined by ELISA. FC and GOS dose-dependently reduced the level of recombinant human CD44 protein (Figure 4A). FOS at 10 ng/mL also reduced the detected levels of CD44; however, its effects were minimal at concentrations lower (1 ng/mL) and higher (100 ng/mL) than 10 ng/mL. Therefore, all three oligosaccharides at appropriate doses can affect CD44 antigenicity. HA was then added to the cells treated with oligosaccharides to observe whether oligosaccharides compete with HA in interacting with CD44. As shown in Figure 4B, HA blocked the inhibitory effect of FC on hypoxia-induced CD44 and phospho-JNK and even promoted the expression of CD44 and phospho-JNK in hypoxic cells but not in normal cells. This finding indicates that the interaction between HA and CD44 induces JNK signal transduction in hypoxic renal tubular cells. The same result was also found in cells treated with FOS or GOS (Figure 4B). Therefore, all three oligosaccharides compete with HA in interacting with CD44 in renal tubular cells.

### 3.5. Oligosaccharides Improve Renal Function and Reduce Inflammation in Mice with Early Stage AKI

**Figure 5:**
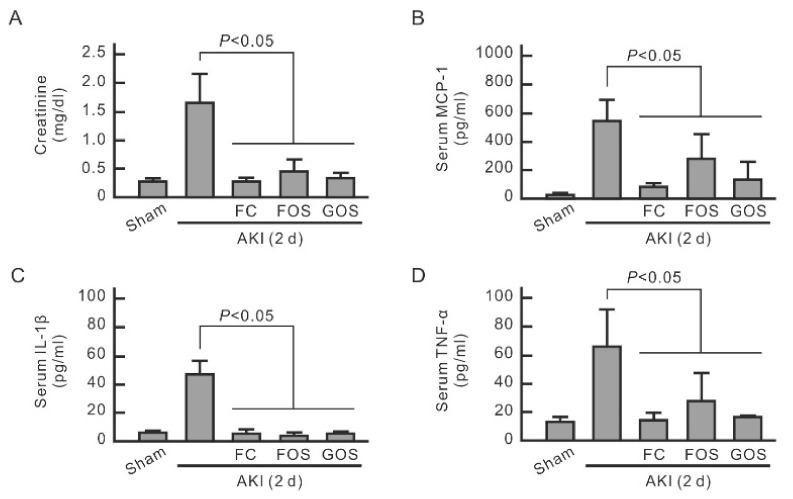
Reducing effect of oligosaccharides on serum creatinine and cytokines in AKI mice at the early stage. Blood was collected from each mouse 2 days after IRI surgery to measure serum creatinine (**A**), MCP-1 (**B**), IL-1β (**C**), and TNF-α (**D**). The results are expressed as means ± SD (n = 8).

**Figure 6:**
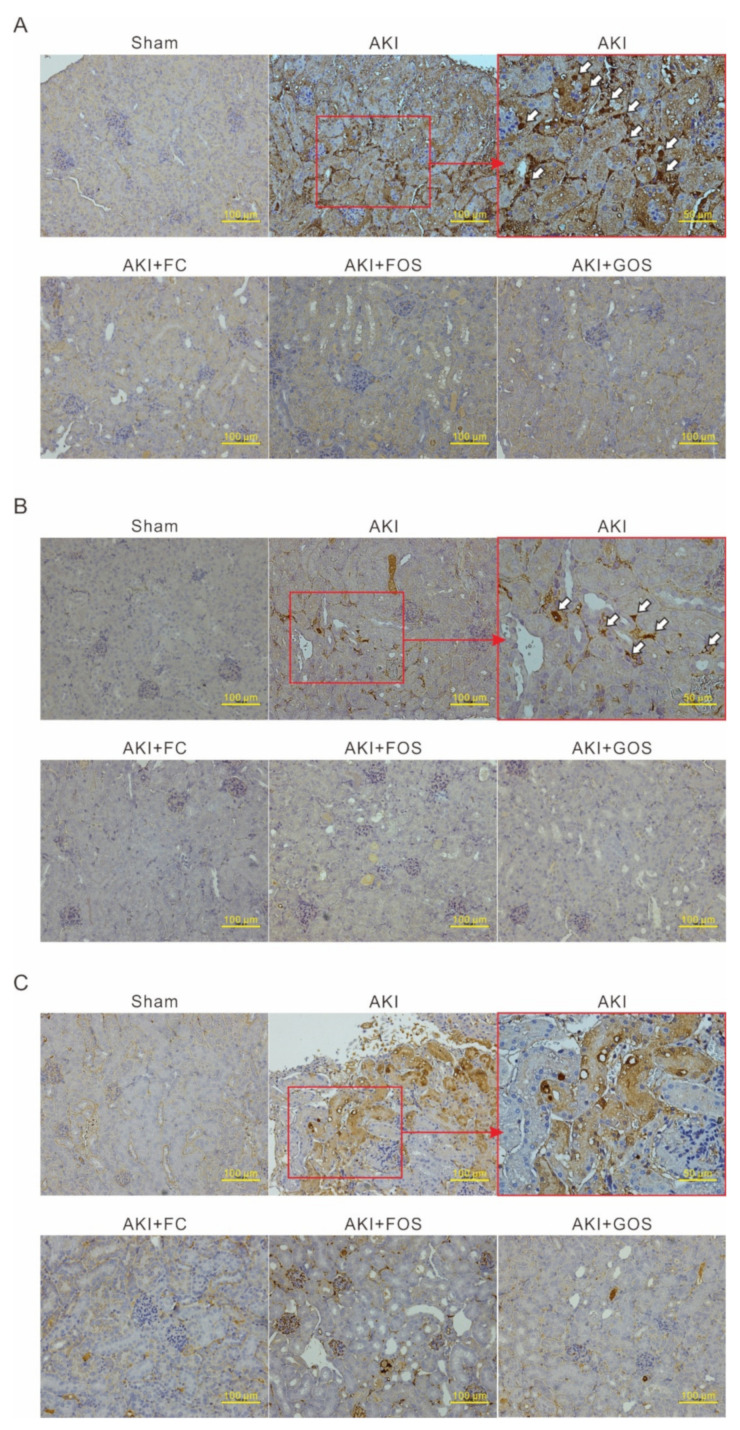
Inhibitory effect of oligosaccharides on renal inflammation in mice with early-stage AKI. The kidneys from each mouse 2 days after IRI surgery were collected for IHC staining. (**A**) Ly6G IHC staining. The white arrow indicates Ly6G positive staining neutrophils. (**B**) F4/80 IHC staining. The white arrow indicates F4/80 positive staining macrophages. (**C**) TNF-α IHC staining.

An AKI mouse model with IRI was established to evaluate the influence of oligosaccharides on renal function and inflammation. Renal function was monitored by detecting serum creatinine. The mice were fed with oligosaccharides only at the initial stage of AKI, i.e., from 1 day before to 2 days after IRI surgery. After this period, serum creatinine significantly increased in AKI mice but not in oligosaccharide-treated AKI mice, indicating that oligosaccharides have improved renal function (Figure 5A). Serum cytokine levels were then monitored to evaluate the inflammation of AKI mice. Serum MCP-1, IL-1β, and TNF-α were significantly increased in AKI mice (Figure 5B–D) but were reduced by all three oligosaccharides. IHC analysis of neutrophil marker Ly6G showed neutrophil infiltration in the kidneys of AKI mice but not in normal mice and oligosaccharide-treated AKI mice (Figure 6A). IHC analysis of macrophage marker F4/80 revealed macrophage invasion in the kidneys of AKI mice but not in normal mice and AKI mice treated with oligosaccharides (Figure 6B). Compared with those of normal mice and AKI mice fed with oligosaccharides, the renal tubules of AKI mice showed high TNF-α expression (Figure 6C). These results indicate that FC, FOS, and GOS reduce renal inflammation in mice with early-stage AKI.

### 3.6. Oligosaccharides Promote Kidney Recovery at Post-AKI

**Figure 7:**
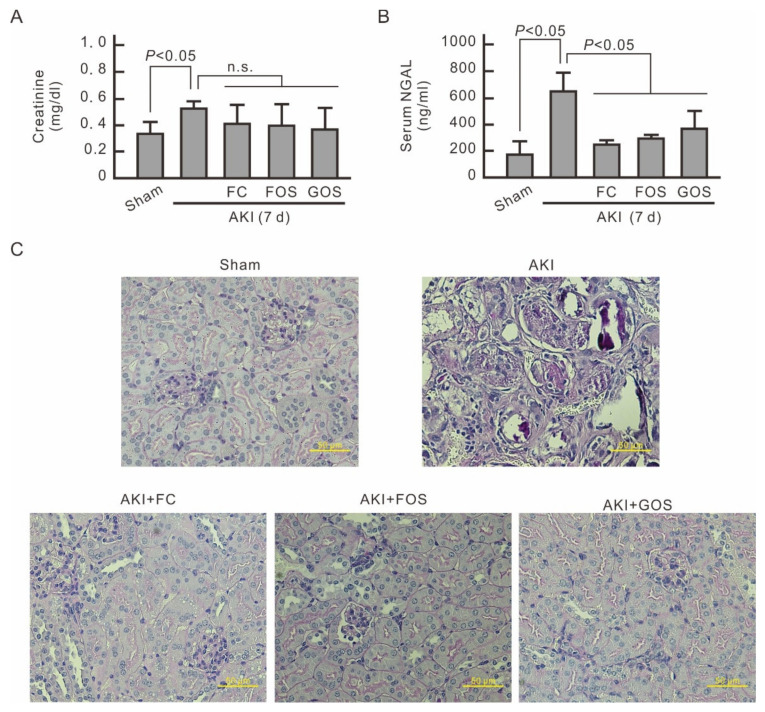
Reducing effect of oligosaccharides on the severity of AKI in post-AKI. Blood and kidneys from each mouse were collected 7 days after IRI surgery for biochemical analysis and IHC staining, respectively. (**A**) Serum creatinine levels. The results are expressed as means ± SD (n = 8). n.s., no significance. (**B**) Serum NGAL levels. The results are expressed as means ± SD (n = 8). (**C**) PAS staining of mouse kidney cortex. Pink staining of brush border is visible in health renal tubules, and loss of brush border is evident in the unhealthy dilated tubules.

On the seventh day after IRI surgery, the serum creatinine of AKI mice decreased but was still higher than that of normal mice (Figure 7A). No significant difference in serum creatinine was observed between AKI mice treated with and without oligosaccharides. However, the level of serum NGAL, a marker of AKI, was still higher in AKI mice than in normal mice and oligosaccharide-treated AKI mice (Figure 7B). This finding indicates that feeding oligosaccharides at the early stage of AKI can reduce renal injury at post-AKI. PAS staining of mouse kidney cortex was conducted at 7 days after IRI surgery. AKI mice exhibited severe tubular injury with tubular dilatation and intraluminal cell debris by tubular necrosis, and AKI mice treated with oligosaccharides showed only minor tubular injury (Figure 7C). Therefore, the intake of FC, FOS, or GOS at the early stage of AKI aids in the recovery of kidney tissues and renal function in AKI mice.

## 4. Discussion

Ischemia is one of the main causes of AKI. Here, a hypoxic cell model and an AKI mouse model with IRI were established to study the influence of oligosaccharides on AKI and their underlying molecular mechanism. Hypoxia induced the expression of MCP-1, IL-1α, and TNF-α in NRK-52E cells, which were inhibited by JNK inhibitor SP600125. CD44 siRNA transfection blocked the hypoxia-induced activation of JNK. These results indicate that hypoxia upregulates CD44, which in turn activates JNK and thereby upregulates cytokines in renal tubular cells. FC, FOS, and GOS reduced CD44 antigenicity and competed with the ligand HA of CD44 of renal tubular cells. This finding implies that the three oligosaccharides can directly interact with CD44 to suppress hypoxia-induced CD44 upregulation, JNK activation, and cytokine expression in renal tubular cells. Although p38 is another important MAP kinase for TNFα and IL-1β during inflammatory responses [21], phosphorylated p38 was not upregulated in hypoxic NRK-52E cells. Therefore, p38 is not involved in cytokine expression in renal tubular cells and the inhibition effect of oligosaccharides on the inflammatory responses of renal tubular cells. FC and GOS upregulated p38 phosphorylation in hypoxic cells, indicating their potential influence on p38-associated inflammatory responses. In animal studies, FC, FOS, and GOS inhibited the increase in serum MCP-1 and IL-1β in mice with early-stage AKI and reduced TNF-α expression in kidney tissues. Cytokines are key effectors of leukocyte recruitment. FC, FOS, and GOS reduced neutrophil infiltration and macrophage invasion in the kidneys of mice with early-stage AKI. At 7 days after surgery, the reduced inflammation and neutrophil infiltration in AKI at the early stage is beneficial for the recovery at the advanced stage. This study reveals for the first time that the consumption of FC, FOS, or GOS at the early stage of AKI can promote recovery by inhibiting kidney inflammation.

To date, the influence of oligosaccharide supplementation on AKI has not been explored. Many oligosaccharides are considered prebiotics and can promote the growth of favorable microbiota [13,22]. Human milk oligosaccharides regulate the development and function of the immune system by constructing specific microbiota [23,24]. Intestinal microbiota is an important modifier of AKI outcome [20]. In theory, oligosaccharides can affect AKI severity by regulating intestinal microbiota. However, microbiota construction by oligosaccharides and immune moderation by microbiota would require more than several weeks. In the current animal study, the mice were fed with oligosaccharides from 1 day before to 2 days after IRI surgery. The results showed the positive effect of oligosaccharides on AKI on the second day after IRI surgery. Therefore, the renal protection of oligosaccharides in the current system is not caused by the influence of microbiota. Many oligosaccharides, including FC, FOS, and GOS, can be absorbed by the body, enter the systemic circulation, and finally be eliminated by the kidneys [14,15,16,22,25]. Those oligosaccharides have a chance to interact with CD44 on renal tubular cells in vivo. By influencing CD44, oligosaccharides can instantly modulate the immune response of the kidney with acute injury, thereby improving its recovery. In addition, human milk oligosaccharides directly modulate immune responses in a local or systemic manner [22,25]. Therefore, the effect of oligosaccharides on immune and other physiological systems is attributed to their direct influences and microbiota changes.

CD44 promotes cell adhesion and acts as a signal receptor in many important physiological phenomena [26]. The interaction of CD44 and its major ligand HA, a major constituent of the extracellular matrix, can enhance cell adhesion, proliferation, migration, and immune responses [27,28,29]. HA binding to CD44 initiates intracellular signaling events by modulating the downstream signaling molecules of CD44, including actin cytoskeleton [30]; ezrin, radixin, and moesin proteins [31]; ankyrin [32]; and non-receptor tyrosine kinase Src [33]. These downstream signal molecules are related to JNK activation. Reorganization of the actin cytoskeleton activates numerous signaling cascades, specifically JNK [34,35]. Ezrin binds with JNK signaling components to facilitate JNK activation in B cells [36]. Ankyrin and Src also induce JNK activation [37,38,39]. Therefore, the signal transduction of CD44 is associated with JNK activation. HA binding to CD44 has been proven to promote JNK activation in breast cancer cells [19]. In the present study, oligosaccharides were found to interfere with the interaction between HA and CD44 to inhibit the downstream JNK signal transduction and consequently the immune response of renal tubular cells. On the basis of these findings, oligosaccharides show potential to suppress other physiological responses related to CD44, especially the proliferation and migration of cancer cells promoted by CD44.

Oligosaccharides currently available on the market mainly include FOS, GOS, FC, and isomaltooligosaccharides [40,41]. FOS, GOS, and FC can be partially absorbed by the body, enter the systemic circulation, and be ingested from vegetables, milk, and seaweed [14,15,16,22,25]. In this work, the influence of these three anionic oligosaccharides on AKI was explored. Although these three oligosaccharides have different monosaccharide compositions, their effects on renal tubular cells under hypoxia are highly similar, including the inhibitory effect on CD44, phosphorylated JNK, and cytokines. Moreover, the added HA interfered with the inhibitory effect of all three oligosaccharides. Therefore, the mechanism of these three oligosaccharides on renal tubular cells is the same regardless of their monosaccharide type. This mechanism may be related to the structural similarities between oligosaccharides and HA. However, the types of monosaccharides in the three oligosaccharides still cause slightly different effects in the current system. FC and FOS reduced JNK expression in hypoxic renal tubular cells, but GOS exhibited a limited effect. The influence of FOS on CD44 antigenicity was weaker than that of FC and GOS. The most effective dose of FOS (0.5 mg/mL) for inhibiting hypoxia-induced CD44 was also higher than that of FC and GOS (0.05 mg/mL). These results suggest that the inhibitory effects of FC and GOS on CD44 signaling transduction are greater than that of FOS. However, animal experiments showed similar renal protective effects from the ingestion of the three oligosaccharides at the initial stage of AKI. Comparing the renal protective effects among the three oligosaccharides in AKI mice is difficult and involves the absorption of oligosaccharides in the intestine. Most oligosaccharides are digested by intestinal bacteria, and only a small portion can be absorbed [42,43]. Digestion efficiency in the intestine varies for different oligosaccharides, which in turn affects their absorption amount. In addition, the length of oligosaccharides also affects the efficiency of their absorption in the gut. In this study, FC with the smallest average degree of polymerization would theoretically be more readily absorbed than FOS with the largest average degree of polymerization. However, animal experiments revealed that after oligosaccharides (10 mg/kg/d) are digested by the intestinal bacteria, a sufficient amount can still enter circulation to achieve a renal protective effect on AKI mice.

In summary, FC, FOS, and GOS directly interact with CD44 to inhibit hypoxia-induced CD44 upregulation, JNK activation, and cytokine expression in renal tubular cells. Ingesting FC, FOS, and GOS at early AKI stages promotes recovery by inhibiting kidney inflammation. AKI suddenly occurs in patients admitted to hospitals and intensive care units, and no treatment has been developed to avoid or ameliorate this condition. On the basis of these findings, the daily ingestion of oligosaccharides can reduce AKI severity and promote kidney recovery in patients with AKI.

## Data Availability

Not applicable.

## References

[1] Makris K., Spanou L. (2016). Acute Kidney Injury: Definition, Pathophysiology and Clinical Phenotypes. Clin. Biochem. Rev..

[2] Silver S.A., Harel Z., McArthur E., Nash D.M., Acedillo R., Kitchlu A., Garg A.X., Chertow G.M., Bell C.M., Wald R. (2018). Causes of Death after a Hospitalization with AKI. J. Am. Soc. Nephrol..

[3] Joannidis M., Druml W., Forni L.G., Groeneveld A.B.J., Honore P.M., Hoste E., Ostermann M., Oudemans-van Straaten H.M., Schetz M. (2017). Prevention of acute kidney injury and protection of renal function in the intensive care unit: Update 2017: Expert opinion of the Working Group on Prevention, AKI section, European Society of Intensive Care Medicine. Intensive Care Med..

[4] Bijuklic K., Jennings P., Kountchev J., Hasslacher J., Aydin S., Sturn D., Pfaller W., Patsch J.R., Joannidis M. (2007). Migration of leukocytes across an endothelium-epithelium bilayer as a model of renal interstitial inflammation. Am. J. Physiol. Physiol..

[5] Bihorac A., Baslanti T.O., Cuenca A.G., Hobson C., Ang D., Efron P.A., Maier R.V., Moore F.A., Moldawer L.L. (2013). Acute kidney injury is associated with early cytokine changes after trauma. J. Trauma Acute Care Surg..

[6] Singbartl K., Formeck C., Kellum J.A. (2019). Kidney-Immune System Crosstalk in AKI. Semin. Nephrol..

[7] Murugan R., Wen X., Keener C., Pike F., Palevsky P.M., Unruh M., Finkel K., Vijayan A., Elder M., Chen Y.-F. (2015). Associations between Intensity of RRT, Inflammatory Mediators, and Outcomes. Clin. J. Am. Soc. Nephrol..

[8] Arana L., Ordoñez M., Ouro A., Rivera I., Gangoiti P., Trueba M., Muñoz A.G. (2013). Ceramide 1-phosphate induces macrophage chemoattractant protein-1 release: Involvement in ceramide 1-phosphate-stimulated cell migration. Am. J. Physiol. Metab..

[9] Kumagai N., Fukuda K., Fujitsu Y., Lu Y., Chikamoto N., Nishida T. (2005). Lipopolysaccharide-Induced Expression of Intercellular Adhesion Molecule-1 and Chemokines in Cultured Human Corneal Fibroblasts. Investig. Ophthalmol. Vis. Sci..

[10] Segerer S., Nelson P.J., Schlöndorff D. (2000). Chemokines, Chemokine Receptors, and Renal Disease: From Basic Science to Pathophysiologic and Therapeutic Studies. J. Am. Soc. Nephrol..

[11] Liang Y., Liang L., Liu Z., Wang Y., Dong X., Qu L., Gou R., Wang Y., Wang Q., Liu Z. (2020). Inhibition of IRE1/JNK pathway in HK-2 cells subjected to hypoxia-reoxygenation attenuates mesangial cells-derived extracellular matrix production. J. Cell. Mol. Med..

[12] Shui H., Gao P., Si X., Ding G. (2010). Mycophenolic acid inhibits albumin-induced MCP-1 expression in renal tubular epithelial cells through the p38 MAPK pathway. Mol. Biol. Rep..

[13] Rastall R. (2010). Functional Oligosaccharides: Application and Manufacture. Annu. Rev. Food Sci. Technol..

[14] Molis C., Flourie B., Ouarne F., Gailing M.F., Lartigue S., Guibert A., Bornet F., Galmiche J.P. (1996). Digestion, excretion, and energy value of fructooligosaccharides in healthy humans. Am. J. Clin. Nutr..

[15] Difilippo E., Bettonvil M., Willems R.H.A.M., Braber S., Fink-Gremmels J., Jeurink P.V., Schoterman M.H.C., Gruppen H., Schols H.A. (2015). Oligosaccharides in Urine, Blood, and Feces of Piglets Fed Milk Replacer Containing Galacto-oligosaccharides. J. Agric. Food Chem..

[16] Kadena K., Tomori M., Iha M., Nagamine T. (2018). Absorption Study of Mozuku Fucoidan in Japanese Volunteers. Mar. Drugs.

[17] Varki A. (2017). Biological roles of glycans. Glycobiology.

[18] Chen C.-H., Sue Y.-M., Cheng C.-Y., Chen Y.-C., Liu C.-T., Hsu Y.-H., Hwang P.-A., Huang N.-J., Chen T.-H. (2017). Oligo-fucoidan prevents renal tubulointerstitial fibrosis by inhibiting the CD44 signal pathway. Sci. Rep..

[19] Chen L., Bourguignon L.Y.W. (2014). Hyaluronan-CD44 interaction promotes c-Jun signaling and miRNA21 expression leading to Bcl-2 expression and chemoresistance in breast cancer cells. Mol. Cancer.

[20] Yang J., Kim C.J., Go Y.S., Lee H.Y., Kim M.-G., Oh S.W., Cho W.Y., Im S.-H., Jo S.K. (2020). Intestinal microbiota control acute kidney injury severity by immune modulation. Kidney Int..

[21] Bradham C., McClay D.R. (2006). p38 MAPK in Development and Cancer. Cell Cycle.

[22] Plaza-Díaz J., Fontana L., Gil A. (2018). Human Milk Oligosaccharides and Immune System Development. Nutrients.

[23] Šuligoj T., Vigsnæs L.K., Abbeele P.V.D., Apostolou A., Karalis K., Savva G.M., McConnell B., Juge N. (2020). Effects of Human Milk Oligosaccharides on the Adult Gut Microbiota and Barrier Function. Nutrients.

[24] Cukrowska B., Bierła J.B., Zakrzewska M., Klukowski M., Maciorkowska E. (2020). The Relationship between the Infant Gut Microbiota and Allergy. The Role of Bifidobacterium breve and Prebiotic Oligosaccharides in the Activation of Anti-Allergic Mechanisms in Early Life. Nutrients.

[25] Rudloff S., Pohlentz G., Borsch C., Lentze M.J., Kunz C. (2012). Urinary excretion ofin vivo13C-labelled milk oligosaccharides in breastfed infants. Br. J. Nutr..

[26] Heldin P., Kolliopoulos C., Lin C.-Y., Heldin C.-H. (2020). Involvement of hyaluronan and CD44 in cancer and viral infections. Cell. Signal..

[27] Lee-Sayer S.S.M., Dong Y., Arif A.A., Olsson M., Brown K.L., Johnson P. (2015). The Where, When, How, and Why of Hyaluronan Binding by Immune Cells. Front. Immunol..

[28] Zöller M. (2011). CD44: Can a cancer-initiating cell profit from an abundantly expressed molecule?. Nat. Rev. Cancer.

[29] Toole B.P., Slomiany M.G. (2008). Hyaluronan: A constitutive regulator of chemoresistance and malignancy in cancer cells. Semin. Cancer Biol..

[30] Marhaba R., Zöller M. (2003). CD44 in Cancer Progression: Adhesion, Migration and Growth Regulation. Histochem. J..

[31] Legg J.W., Lewis C.A., Parsons M., Ng T., Isacke C. (2002). A novel PKC-regulated mechanism controls CD44–ezrin association and directional cell motility. Nat. Cell Biol..

[32] Lokeshwar V.B., Fregien N., Bourguignon L.Y. (1994). Ankyrin-binding domain of CD44(GP85) is required for the expression of hyaluronic acid-mediated adhesion function. J. Cell Biol..

[33] Bourguignon L.Y.W., Zhu H., Shao L., Chen Y.-W. (2001). CD44 Interaction with c-Src Kinase Promotes Cortactin-mediated Cytoskeleton Function and Hyaluronic Acid-dependent Ovarian Tumor Cell Migration. J. Biol. Chem..

[34] Ispanovic E., Haas T. (2006). JNK and PI3K differentially regulate MMP-2 and MT1-MMP mRNA and protein in response to actin cytoskeleton reorganization in endothelial cells. Am. J. Physiol. Physiol..

[35] Benoit B., Baillet A., Poüs C. (2021). Cytoskeleton and Associated Proteins: Pleiotropic JNK Substrates and Regulators. Int. J. Mol. Sci..

[36] Parameswaran N., Enyindah-Asonye G., Bagheri N., Shah N.B., Gupta N. (2013). Spatial Coupling of JNK Activation to the B Cell Antigen Receptor by Tyrosine-Phosphorylated Ezrin. J. Immunol..

[37] Harada T., Matsuzaki O., Hayashi H., Sugano S., Matsuda A., Nishida E. (2003). AKRL1 and AKRL2 activate the JNK pathway. Genes Cells.

[38] Holzer R.G., Park E.-J., Li N., Tran H., Chen M., Choi C., Solinas G., Karin M. (2011). Saturated Fatty Acids Induce c-Src Clustering within Membrane Subdomains, Leading to JNK Activation. Cell.

[39] Poon C.L., Brumby A.M., Richardson H.E. (2018). Src Cooperates with Oncogenic Ras in Tumourigenesis via the JNK and PI3K Pathways in Drosophila epithelial Tissue. Int. J. Mol. Sci..

[40] Tingirikari J.M.R. (2018). Microbiota-accessible pectic poly- and oligosaccharides in gut health. Food Funct..

[41] Shang Q., Shan X., Cai C., Hao J., Li G., Yu G. (2018). Correction: Dietary fucoidan modulates the gut microbiota in mice by increasing the abundance of Lactobacillus and Ruminococcaceae. Food Funct..

[42] Yang J., Maldonado-Gómez M.X., Hutkins R.W., Rose D.J. (2014). Production and in Vitro Fermentation of Soluble, Non-digestible, Feruloylated Oligo- and Polysaccharides from Maize and Wheat Brans. J. Agric. Food Chem..

[43] Moon J.S., Shin S.Y., Choi H.S., Joo W., Cho S.K., Li L., Kang J.-H., Kim T.-J., Han N.S. (2015). In vitro digestion and fermentation properties of linear sugar-beet arabinan and its oligosaccharides. Carbohydr. Polym..

